# Anthracyclines as Topoisomerase II Poisons: From Early Studies to New Perspectives

**DOI:** 10.3390/ijms19113480

**Published:** 2018-11-06

**Authors:** Jessica Marinello, Maria Delcuratolo, Giovanni Capranico

**Affiliations:** Department of Pharmacy and Biotechnology, University of Bologna, via Selmi 3, 40126 Bologna, Italy; mdelcuratolo@gmail.com

**Keywords:** topoisomerase II, anthracyclines, DNA damage, toxic effects, immunogenic cell death

## Abstract

Mammalian DNA topoisomerases II are targets of anticancer anthracyclines that act by stabilizing enzyme-DNA complexes wherein DNA strands are cut and covalently linked to the protein. This molecular mechanism is the molecular basis of anthracycline anticancer activity as well as the toxic effects such as cardiomyopathy and induction of secondary cancers. Even though anthracyclines have been used in the clinic for more than 50 years for solid and blood cancers, the search of breakthrough analogs has substantially failed. The recent developments of personalized medicine, availability of individual genomic information, and immune therapy are expected to change significantly human cancer therapy. Here, we discuss the knowledge of anthracyclines as Topoisomerase II poisons, their molecular and cellular effects and toxicity along with current efforts to improve the therapeutic index. Then, we discuss the contribution of the immune system in the anticancer activity of anthracyclines, and the need to increase our knowledge of molecular mechanisms connecting the drug targets to the immune stimulatory pathways in cancer cells. We propose that the complete definition of the molecular interaction of anthracyclines with the immune system may open up more effective and safer ways to treat patients with these drugs.

## 1. Introduction

The clinical use of DNA topoisomerase inhibitors is vast in oncology as they are approved for first-line chemotherapy for several solid and hematological malignancies. Among them, the main drug classes are anthracyclines, epipodophyllotoxins, and camptothecins: the parent compounds were discovered between 1960 and 1970 of the last century [1], and since then the research for better analogs or new agents has been very intense. Nevertheless, we must admit that the parent drugs or initially-developed analogs are still used in medical oncology. In more recent years, several biologicals, mainly antibodies, have been developed with effective therapeutic activity, providing a striking turn-around for cancer patient treatments [2]. Moreover, the recent combined development of personalized medicine with more accessible individual genomic data and of immune checkpoint inhibitors further promises to change significantly the therapy of human cancers [3,4]. In this new context, one important question is whether anthracyclines, and other topoisomerase II poisons, will continue to be an important asset for oncologists. Here, we present a historical view of the molecular mechanism of anthracyclines as DNA topoisomerase II poisons, and discuss diverse findings that need to be understood at molecular and cellular levels in a consistent framework for further developments of anthracycline drugs in modern medical oncology.

## 2. Human Top2α and Top2β are the Cellular Targets of Anthracyclines

**Figure 1:**
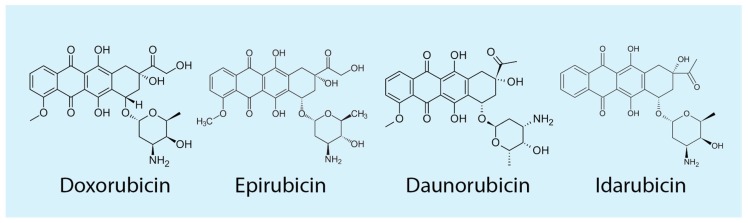
Chemical structures of the most clinically-used anthracyclines.

Anthracyclines (Figure 1) are highly effective poisons of Top2 in eukaryotic cells, therefore the biological effects of the drugs are affected by enzyme functions and activities [5]. Human DNA topoisomerases are classified into two classes based on structure and mechanisms. Monomeric type I enzymes (Top1) catalyze the formation of DNA single-strand breaks during the catalytic cycle, whereas dimeric type II enzymes (Top2) introduce double-strand breaks (DSBs) in the DNA template [5,6,7,8,9]. Catalytic activity of Top2 is mediated through a unidirectional strand-passage mechanism by which one DNA segment is driven through a DNA cut resulting in DNA relaxation, negative DNA supercoiling, knotting/unknotting, and catenation/decatenation activities depending on the specific enzyme. Top2 poisons, including anthracyclines, block the catalytic enzyme reaction stabilizing an intermediate wherein a DNA strand is cut and covalently linked to the enzyme. As the DNA is broken, this reaction intermediate is called a DNA-Top2 cleavage complex (Top2cc), which may eventually lead to cell apoptosis when DNA cuts become irreversible at genomic regions of active DNA synthesis. Thus, topoisomerase II-targeting anticancer anthracyclines increase Top2-mediated irreversible DNA damage preferentially in proliferating cancer cells as compared to post-mitotic normal cells. Further discussions of enzyme structures and catalytic mechanisms, and mechanisms of topoisomerase poisons can be found in several recent reviews [5,10,11,12,13,14].

Two isoforms of Top2 are present in mammals, Top2α and Top2β, the genes of which are localized at chromosomes 17 and 3, respectively [10,15]. First evidence of the existence of a second human Top2 (Top2β) were published in 1987, when distinct isoforms of Top2 were isolated [16], and in 1990 when partial cloning of Top2β gene from HeLa cells was reported [17]. A few years later, the complete coding region was cloned and the recombinant Top2β was purified [18,19,20]. Human Top2α and Top2β show almost 70% homology in their amino acid sequences, mainly at the N-terminal and central catalytic domains, whereas the major sequence divergence involves the C-terminal domain [10,15].

The functions of the two enzymes are different in cells, and here we only summarize the information relevant to the biological effects of anthracyclines. First evidence suggesting that the two isoforms have different functions in mammalian cells came from gene expression studies in normal murine tissues during development [21], where Top2β was found expressed almost in all tissues whereas Top2α gene expression was limited to tissues with a high fraction of cell proliferation. Top2α is indeed involved in the replication process with a crucial role in the separation of DNA helices and in chromosome compaction and segregation during mitotic divisions [5,10,22]. Consistently, Top2α gene is highly expressed in S and G2 cell cycle phases, and is essential in eukaryotes, including mammals as the lack of Top2α in mice leads to a very early arrest of cell division during embryonic development [23]. As Top2α has an essential role for the disentanglement of daughter DNA helices, other reports showed that it can interact with cohesins and SMC proteins to maintain chromatid and chromosome structures [5,10]. In particular, Christensen et al. [24] demonstrated that, while the interphase is characterized by the accumulation of both Top2α and Top2β in the nuclear compartment, particularly in nucleoli, at mitosis only Top2α becomes tightly associated with chromosomes. A recent report then showed that Top2α causes the axial shortening of chromosomes and, along with condensin and the chromokinesin KIF4, can shape chromosome morphology at mitosis [25]. The specific mitotic role of Top2α vs. Top2β is likely determined by the highly divergent *C*-terminal domain as it has been demonstrated that substituting this domain of the β isoform with that of the α isoform produces an enzyme chimera that behaves as Top2α [26]. Interestingly, when the α isoform is forced outside the nucleus by localization signal mutations, the β isoform cannot be recruited to condensing chromatin, thus suggesting that Top2β cannot rescue a loss of Top2α for chromosome condensation at mitosis [22,27]. Thus, Top2α is considered a marker of cell proliferation, and expression studies in normal human tissues and cancers have shown that it is often overexpressed in aggressive or rapidly proliferating tumors, while it is undetectable in differentiated and quiescent cells [28,29]. Top2β is instead ubiquitously expressed at significant levels in normal tissues and cancers, however it is generally more expressed in proliferating cells and tumors [28,30].

The roles of Top2 isoforms in transcription and transcription regulation is an active research area. Either Top2 and Top1 can relax torsional stress of DNA template during transcription to allow RNA polymerase elongation [5,31], however they are recruited at different regions of active genes such as promoters or along the entire gene, respectively [32]. While Top1 is generally needed at all transcribed genes, Top2 has been proposed to be required especially at highly active genes to resolve the high rate of torsional stress generated by RNA polymerases [32]. In addition to this basic topology-related function, Top2 isoforms have been proposed to have specific functions in transcription regulation at different developmental stages.

A burst of Top2β gene expression was reported in mouse brains immediately after birth suggesting a critical role of the β isoform in brain development [21], which was further supported by a later paper showing an alteration of neuronal development in brain-specific Top2β depleted mice [33]. In addition, Top2β knockout mice exhibited perinatal death due to severe neuronal defects affecting their respiratory tracts [23,34]. Successive microarray analyses of Top2β-knockout mouse brains demonstrated recruitment of this enzyme at gene promoters at late stages of neuron differentiation [35]. Top2β was also found to bind H3K4-dimethylated promoters of actively transcribed genes in mouse neuronal cells [36], and in particular it was associated with the expression of long genes [37]. Another report showed that Top2α is preferentially recruited at promoters of transcribed genes in murine embryonic cells [38]. Interestingly, many housekeeping genes are targets of both Top2α and Top2β in embryonic and terminal differentiated cells, respectively, while unique gene targets have functions in pluripotency and neurogenesis pathways, respectively. Top2α activity is also critical for high transcription rates of major ribosomal RNA genes (rDNA) in growing cells as it interacts with the transcription factor RRN3, which recruits RNA polymerase I to rDNA promoter along with Top2α [39]. In growing cells, the activation of rDNA transcription is accompanied by transient DNA cleavage at the promoter, which is dependent on Top2α, suggesting that this enzyme can modulate DNA topology at the rDNA promoter which is needed for pre-initiation complex formation [39].

In terminal differentiated cells, transient DNA cleavage mediated by Top2β has been proved to occur at certain promoter regions and to be required for transcriptional gene activation. Interestingly, Chd7 (chromodomain helicase DNA-binding protein 7) is needed along with Top2β for the expression of long neuronal genes in granule neurons of mouse cerebellum revealing a Top2β-dependent pathway of chromatin remodeling for cell-specific gene expression [40]. Madabhushi et al. demonstrated that etoposide, a poison of Top2, could lead to the up-regulation of the early-response genes Fos and Npas4 in neuronal cells [41] and that Top2β is enriched by ChIP analyses at gene promoter region [41,42]. Moreover, the report showed reduced levels of double-strand breaks at the Fos promoter of neurons with decreased Top2β levels upon NMDA (*N*-methyl-d-aspartate) stimulation suggesting that Top2β is required for double-strand breakage formation for rapid gene activation [41]. Similarly, transient double-strand breaks by Top2β were also observed at the pS2 promoter upon 17β-estradiol (E2) stimulation and required for transcriptional activation of target genes [43]. ChIP analyses show that Top2β recruitment sites are enriched in gene promoters and that E2 can modulate the profiles of Top2β-binding sites modulated in the genome [44]. Thus, Top2β activity may have a specific role in transcription regulation of specific genes in terminal differentiated neurons, raising the question of which are the factors driving the recruitment of the enzyme to chromatin sites.

Recently, a transcription-independent Top2β activity was shown to occur at anchor sites of topologically-associated domains (TADs). Genomic maps of Top2β binding sites in chromatin of interphase cells showed that this isoform was associated not only with active genes but also with CTCF/cohesin-bound chromatin regions close to TAD boundaries [45]. By genome mapping of Top2β-mediated double-stranded breaks, it has been shown that the β isoform can promote breakage at chromatin-bound sites in interphase cells. Interestingly, these DNA breaks are concentrated at loop anchors of TADs along with the presence of CTCF and Rad21, a subunit of the cohesin complex [46]. These reports suggest that Top2β can resolve DNA torsional stress of TADs acting at loop anchor regions. Interestingly, those regions have been associated with sites of DNA translocations frequently found in secondary cancers developed after a chemotherapy regimen containing Top2 poisons such as anthracyclines and etoposide [45,46] (see also below).

## 3. Top2 Poisoning Activity of Anthracyclines

It is widely accepted that the effective anticancer activity of anthracyclines is due to the drug cell killing activity that is specific for proliferating cancer cells. A vast amount of published data demonstrates that the cellular target of anthracyclines, relevant for their therapeutic activity, is Top2 [47,48,49]. These drugs interfere with both human Top2 isoforms [50]: the drug action on Top2α is generally considered the molecular basis of anthracycline activity as this isoform has a main role during replication and cell proliferation [13,51]. In contrast, Top2β has been associated with long-term side effects of anthracyclines such as cardiotoxicity and secondary malignancies (see below), however Top2β may contribute to cell killing activity as well [15,50,52].

The mode of action of anthracyclines (Figure 1) in interfering with either Top2 isoforms is very similar, as shown by structural, biochemical and cellular studies [12,51,53]. The drugs are very potent and efficient in stabilizing Top2cc [47,48,54], resulting therefore in highly lethal DNA breaks in proliferating cancer cells [55,56]. Initial findings clearly showed that anticancer Top2 poisons of different structural families induce DNA cleavage in a sequence-selective manner, which can be revealed experimentally as drug-specific cleavage intensity patterns in sequencing gels [47,48,57]. To establish the mode of action of anthracyclines, early studies investigated the distribution and nucleotide sequence environments of doxorubicin-enhanced sites in defined DNAs, such as SV40 genome with purified Top2. Distributions of DNA cleavage sites induced by Top2 in the presence of diverse drugs resulted in distinctive patterns of enhanced cleavage. DNA cleavage usually occurred on both DNA strands with the expected four base-pair 5’ stagger and strong sites tended to occur within AT-rich tracts such as the major nuclear matrix-associated SV40 DNA [58]. In contrast to other drugs, such as etoposide, doxorubicin-specific sites were found to be most concentrated at A/T runs of in-vitro studied DNA fragments. Interestingly, cleavage intensities changed with time depending either on the site or on the drug, suggesting that Top2 can move along the DNA from a kinetically- to a thermodynamically-preferred site [58]. A striking finding was that among doxorubicin-stabilized sites, none coincided with any of the Top2 II cleavage sites observed without drugs [59]. DNA cleavage at enzyme-only sites declined over time with doxorubicin and was never stimulated by the drug. The sequence selectivity was due to the doxorubicin requirement for an A at the 3’ terminus of at least one of the two strand breaks produced by Top2. Conversely, none of the enzyme-only sites had an A simultaneously at 3’ termini of the DNA cleavage. Thus, the findings showed that 3’-A requirement for anthracycline-enhanced DNA cleavage was not compatible with Top2-only cleavage explaining the reciprocal exclusivity of the two site sets, which was likely due to a different thermodynamic equilibrium [59].

Different Top2 poisons have different nucleotide requirements at positions adjacent to the strand break, including −2, −1, +1, and +2 positions, leading to a classification of structurally-unrelated poisons in distinct functional classes [53,60,61,62,63]. This knowledge was based on different experimental approaches: (i) statistical analyses of hundreds of DNA cleavage sites promoted in vitro by Top2 in the presence of different agents; (ii) mutational studies of cleavage sites, and (iii) cross-linking of photo-inducible analogs [62,64,65]. Altogether these findings led to a common molecular model of the action of Top2 poisons: a Top2 poison binds at the interface of the enzyme/DNA complex forming a ternary complex wherein the poison binds to its receptor at the site of DNA cleavage impeding strand religation and interacting with both DNA bases and active site amino acid residues of Top2 [59,64,66]. Different compounds likely interact differently with the receptor site thus explaining the DNA site selectivity resulting in specific cleavage intensity patterns in sequencing gels [67]. Thus, altogether these findings showed that Top2 poisons are peculiar compounds as their target is a binary enzyme-DNA complex rather than each component alone [53]. This model was then expanded further leading to the “interfacial inhibitor” model [68]. Recent X-ray crystal investigations added the last and final evidence of the model as Top2 poisons were shown to be placed at the predicted site [69]. During the following years, this knowledge led to several attempts to rationally design either hybrid poisons with effective pharmacological activity, fusing for instance structural determinants of etoposide with those of DNA intercalating agents or dual-enzyme poisons directed against both Top2 and Top1 [70,71]. Interestingly, these attempts are still very active in the field [72,73], and may eventually lead to a better anticancer topoisomerase poison in the future.

Doxorubicin and other anthracycline analogs have very peculiar Top2-related effects at cellular levels. Dose response curves of DNA damage induced by doxorubicin demonstrated a dose-dependent level of DNA damage but up to a certain concentration only, as at higher concentrations DNA damage levels dropped to control levels [54,74,75]. Therefore, these bi-phasic dose-response curves are due to the direct action of anthracyclines on Top2cc as very similar curves were observed in in-vitro DNA cleavage assays using purified enzymes [47,48]. This phenomenon is peculiar to anthracyclines and other high-affinity DNA intercalating agents, and is due to the strong affinity of the compound for DNA duplexes that prevents Top2 from binding to DNA. Interestingly, in living cancer cells DNA cleavage levels are progressively reduced over time when doxorubicin is removed from the culture medium, however when doxorubicin concentration was high, DNA cleavage levels started to increase upon drug removal from the medium [75]. This was somewhat surprising but it can be understood by the specific dual action of anthracyclines: strong binding to both Top2cc and DNA duplexes. These peculiar aspects of cellular drug activity must be considered when investigating the molecular mechanisms and pathways activated by anthracyclines or other strong DNA binders.

Few investigations have been published on the sequence specificity of Top2 poisoning by anthracyclines in chromatin of living cells. This is due to the general belief that Top2-mediated DNA cleavage is determined by the enzyme with little influence of the chemical used. However, different poisons promoted DNA cleavage by Top2 at different sequences in in vitro systems and in living cells. Using *D. melanogaster* Kc cells, an anthracycline analog, clerocidin and VM-26 (a VP-16 analog) were shown to have highly different cleavage sequence patterns at transcriptionally-active and -silent chromatin [76,77,78]. These reports revealed that Top2 could be localized to promoter of histone genes only with two poisons (anthracyclines and clerocidin) while VM-26 was ineffective in localizing Top2 at these particular genomic sites. The results thus showed that a loose sequence specificity of poisons can become a determinant of cleavage localization in chromatin as the presence of nucleosome can markedly restrict the accessibility of DNA to Top2 [79].

## 4. Cardiotoxicity and Secondary Cancers Caused by Anthracyclines

The production of reactive oxygen species in heart cell mitochondria has often been proposed as a molecular base of drug cardiac toxicity [80]. It is argued that when drugs reach a high concentration in the blood of patients, the generation of reactive oxygen species becomes significant and constitutes the main cause of damage to cardiomyocytes that heavily depend on mitochondria energy metabolism. However, other findings argue against a significant role of oxygen radicals in anthracycline clinical effects. Both Top2α and Top2β are transported into mitochondria of mammalian cells [81], however in cell tissues that do not express Top2α, such as terminal differentiated cardiomyocytes, only the β isoform is present. This knowledge led to investigations of the role of Top2β in anthracycline cardiotoxicity. In 2007, Liu et al. demonstrated γH2AX induction in H9C2 cardiomyocytes after doxorubicin treatment in a dose-dependent manner with high levels of DNA damage observed at low concentration of drug [82]. DNA damage by doxorubicin was likely due to the β isoform as MEF cells depleted of Top2β exhibited reduced γH2AX levels and sensitivity to doxorubicin [82]. In a mouse model of cardiomyocyte-specific deletion of Top2β gene, the lack of Top2β in heart cells was shown to protect mice from doxorubicin-induced heart cell damage and development of progressive heart failure [83]. The tissue-selective deletion of Top2β gene did not impair mice life or heart functions, suggesting that Top2β is not required for normal homeostasis of adult hearts. Transcriptome analyses showed down-regulation of proapoptotic genes in Top2β-depleted cardiomyocytes after doxorubicin treatment. Doxorubicin caused major alterations of mitochondria functionality in WT hearts whereas mithocondrial dysfunctions were much reduced in Top2β knockout cardiomyocytes [83]. These drug effects can lead to an increase of reactive oxygen species, which is likely a consequence rather than the cause of mitochondria dysfunction following doxorubicin poisoning of Top2β in mitochondria. Thus, the knowledge that Top2β is the cellular target responsible for heart failures caused by anthracyclines is a strong rational for the discovery and development of new anthracycline analogs (in general, new Top2 poisons) more specific for Top2α than Top2β (see below).

Top2-mediated DNA cleavage has long been suspected to cause chromosome translocations that can lead to oncogene activation and secondary cancers in patients treated with Top2 poisons for a primary cancer [84]. Secondary cancers after a primary cancer-related therapy have become a concern as cancer survivors have an increased risk of secondary tumors. A recent review has shown that childhood cancer survivors have more than two-fold increased risk for acute leukemia/myelodysplasia and solid tumors after the age of 40 [85]. Beyond radiation, a well-studied cause of secondary cancers, alkylating agents and Top2 poisons (etoposide, doxorubicin and mitoxantrone) have the best-established association with secondary cancers. In particular, anthracyclines are associated with acute leukemia/myelodysplasia and solid tumors including breast cancers and sarcoma [85].

Top2β, but not Top2α, appears to play a main role in the increased cancer incidence in patient survivors. In a mouse model of skin melanoma induced by etoposide, the skin-specific deletion of Top2β gene has been shown to protect skin cells from cancer transformation [81]. Consistently, it has been shown that the Top2β poison induced DNA damage and genome rearrangements, which were dependent on proteolysis of Top2βccs [86]. Secondary acute myeloid leukemias in patients are often characterized by balanced translocations involving the mixed lineage leukemia (MLL) locus at chrm11q23, which most often occurs at a 8-kb breakpoint cluster region (BCR) [84]. Interestingly, the MLL BCR share distinct DNA and chromatin features with BCRs of other genes involved in chromosomal translocations found in secondary leukemia [84]. These features include matrix-attachment sequences, CTCF binding, DNaseH1 hypersensitivity, specific histone patterns, and Top2 DNA cleavage sites [87,88,89]. As chromosome translocations can be due to non-homologous joining of DNA ends belonging to different chromosome, it is interesting to note that Top2 DNA cleavage sites are very close to translocation sites detected in secondary acute leukemia [84,88]. As broken DNA ends should be close to each other to be joined by DNA repair mechanisms, the proximity of expressed MLL and partner genes has been investigated in interphase cells [90]. Cowell et al. demonstrated that the transcribed MLL gene is often in close proximity or even in the same transcription factory to one of the translocation partner genes. In addition, etoposide-induced DNA cleavage and genomic instability are markedly dependent on Top2β but not Top2α isoforms. Thus, the findings suggest that an illegitimate end-joining repair event can occur as two partner genes are transcribed in the same factory and are cleaved at their BCRs by etoposide-stabilized Top2βcc [90]. Interestingly, BCRs of MLL and other partner genes have chromatin characteristics in common with “loop anchors” of TADs (see above) where Top2β has been shown to be active and to modulate DNA topology of chromatin domains [46].

Thus, therapy-related chromosomal translocations causing secondary cancers can be due to poison interference with Top2β rather than Top2α. Several studies on the mechanisms of secondary cancers have focused on etoposide, and not anthracyclines, however the findings are relevant for all clinically used Top2 poisons as they are not specific for one isoform. Future studies will establish the precise mechanisms in the case of doxorubicin and other drugs. Current knowledge also emphasizes the importance of developing Top2α-specific poisons as anticancer drugs to avoid the cardiomyopathies and secondary cancers caused by anthracyclines and other Top2 poisons.

## 5. Current Attempts to Improve the Clinical Efficacy of Anthracyclines

To overcome doxorubicin-induced cardiomyopathy in cancer patients, dexrazoxane (ICRF-187 or Zinecard) has been approved and is currently used in combination with doxorubicin for metastatic breast cancer patients that have been treated already with ≥300 mg/m^2^ of doxorubicin [91]. Dexrazoxane is a Top2 catalytic inhibitor as it is able to bind to the ATPase domains in the *N*-terminal region and to block enzyme activity preventing the start of the catalytic cycle [82,92]. Liu et al. [82] demonstrated the effects of dexrazoxane on DNA damage and Top2β stability in heart cells. Attenuated effects on DNA damage levels by dexrazoxane were observed in H9C2 cardiomyocytes after doxorubicin treatments suggesting the implication of Top2β in the protective effect of dexrazoxane. In contrast, no change was shown in γH2AX signal after CPT treatment alone or in combination with dexrazoxane. Incubation with dexrazoxane was demonstrated to induce proteasomal degradation of Top2β but not Top2α in cardiomyocytes preventing DNA damage formation after doxorubicin treatment [82].

Catalytic inhibitors of Top2 can therefore be used to avoid toxic effects of anthracyclines and several attempts have been made to develop isoform-specific Top2 inhibitors ([93] and references therein). However, Top2β-specific inhibitors could be even better compounds as they would not affect Top2α. In contrast, to optimize the antitumor activity, current research efforts aim at the discovery of Top2α-specific poisons, which are expected to not induce cardiomyopathies and secondary cancers, likely due to the Top2β. Structural enzyme similarities of human Top2 isoforms make difficult the discovery of isoform-specific small molecules acting as poisons, however screening programs can find isoform-specific agents [94]. Interestingly, a synthetic phenanthridine alkaloid (NK314) has been shown to act as a Top2α-specific poison in cancer cells [95]. This compound can increase Top2αccs, but not Top2βccs, in cultured cells and gene deletion of the former, but not the latter, confers cell resistance to NK314 suggesting that Top2α is the cellular target of this compound in vivo. NK314 has been shown to act as a dual inhibitor of Top2α and DNA-dependent protein kinase [96], suggesting that the cell killing activity is potentiated by targeting two enzymes. Interestingly, pixantrone, a compound structurally-close to anthracyclines, has been shown to target more effectively Top2α than Top2β in living cancer cells [97].

Moreover, anthracycline-based agents or complexes have been developed with the common aim of reducing the toxic effects and improving the therapeutic index. This could be achieved by (i) the development of molecules with new structure; (ii) drugs or prodrugs conjugated to selected antibody or loaded on nanostructures for specific tumor targeting. Here, we summarize recent advances related to these two general attempts.

### 5.1. Discovery of New Analogs

Modifications of the sugar moiety (Figure 1), a structural determinant for Top2 poisoning and anticancer activities of anthracyclines, have been attempted to enhance target recognition. In particular, disaccharide analogs have been developed and their cell killing and anticancer activities were specifically dependent on reciprocal spatial orientation of sugar monomers. The results reveal an important role for the second (non-DNA interacting) sugar in drug activity, however the pharmacological effects of disaccharide analogs could not be fully explained by interference with Top2 [98]. Interestingly, parent drugs and disaccharide derivatives have been reported to remove nucleosomes (nucleosome eviction) at open chromatin regions with consequences on the epigenetic regulation of transcription and reduced DNA repair of double-strand breaks caused by the drugs [99]. The new mechanism is specific for anthracyclines as other Top2 poisons do not share the effect on nucleosomes, and the drug-altered nucleosome remodeling may explain at least partially the resistance of acute myeloid leukemia cells to anthracyclines [100]. Whether this new mode of action of anthracyclines contributes to the biological drug activities needs to be fully established with further studies.

The topopyrones are interesting fungal products with an anthraquinone–polyphenolic structure, very similar to the anthracycline planar ring moiety, which act as poisons of both Top1 and Top2. Zaleski et al. [72] performed structure–activity studies to define which orientation of the fused 1,4-pyrone ring and halogen substituents contribute to the Topoisomease poisoning activity of Topopyrones. Therefore, they defined a pharmacophore able to stabilize both Top1cc and Top2αcc. Other compounds that have been demonstrated to be dual Top1 and Top2 inhibitors are the Ruithenim–anthraquinone complexes synthesized by Kou et al. [73].

Shchekotikhin et al. [101] worked on anthra[2,3-b]furan-3-carboxamides and found that some compounds are dual Top1/Top2 inhibitors. These agents form stable intercalative complexes with duplex DNA and attenuate Top1 and Top2 relaxation activity with a mechanism that is probably different from poisons. Other Top2 inhibitors, structurally similar to anthracyclines, that do not act as poisons are bisanthrapyrazole compounds containing piperazine linkers synthesized by Zhang et. al. [102]. These derivatives strongly inhibit the decatenation activity of Top2α without Top2cc stabilization and can also block the relaxation activity of Top1.

### 5.2. Specific Delivery of Anthracyclines

To improve drug solubility and stability, extend drug half-lives, increase drug concentrations at cancer tissues, a wide research activity has focused on the optimization of drug delivery with liposomes, functionalized nanoparticles, dendrimers or micelles [103]. Several groups have reported many combinations of drug, loaded on different nanosystems, which were tested in vitro and in vivo. Here, we summarize recent reports related to anthracyclines.

Ke et al. [104] incorporated into the surface of doxorubicin loaded liposomes, eight repeated sequences of aspartate (Asp8) and folate, obtaining a dual-targeting liposomal system in which Asp8 target preferentially the resorption surface of bones and folate target the tumor cells. They nicely demonstrated by in vivo distribution imaging and binding assay, that the system has a strong bone targeting effect and high cytotoxicity, associated with a prolonged blood circulation time which favored drug accumulation in the tumor. This system could potentially be used in bone metastasis of breast cancers as normal doxorubicin uptake is poor in such metastatic sites due to bone microstructures.

A promising approach to specifically target cancer tissues is based on the conjugation between monoclonal antibody and anticancer drugs. CD147 is localized on the surface of tumor cells but not in normal tissue and promotes tissue invasion by cancer cells. Asakura et al. [105] took advantage of the antigen to test the therapeutic effect of anti-CD147-labeled polymeric micelle-encapsulated with a conjugate of doxorubicin and glutathione (GSH-DXR), which they previously demonstrated to be more potent compared to doxorubicin alone. Micelles accumulated quickly in cancer cells, in a manner dependent on CD147 expression, showing a specific and highest cytotoxicity in CD147-expressing carcinoma cells. The data suggest that the drug-conjugated micelles could serve as an effective delivery system in CD147-expressing tumors. Madhankumar et al. [106,107] focused on IL13Rα2, a cancer associated receptor with an important role in tumor cell migration, invasion, and anti-apoptotic activity. They looked for IL13Ra2 presence in several carcinoma tissues and tested liposomal IL13-conjugated doxorubicin against glioblastoma tumors and in malignant peripheral nerve sheath tumors, demonstrating a decrease of tumor burden both in vitro and in vivo [108].

Santiago et al. [100,109] immobilized gemcitabine and doxorubicin on gold nanoparticles through a pH-sensitive amide bond, which allows the release of drugs only at acidic pH, a characteristic of cancer cells. In addition to this system, they also modified these nanoparticles adding the folate, obtaining a targeted controlled-release delivery of the drug, reducing the side effects, and increasing the efficacy. Han et al. [110] developed a new nanocarrier that delivered doxorubicin combined with rhein to suppress progression of human ovarian cancer cells with drug resistance, while Xu et al. [111] loaded mitoxantrone (structurally similar to antharcyclines) and verapamil in polysaccharide-based nanoparticles to overcome multidrug resistance in breast tumor. Other two recent works have provided interesting results. Wu et al. [112] developed a lipid-coated hollow calcium phosphate nanoparticle for the combined application of doxorubicin and paclitaxel to human lung cancer A549 cells. Shi et al. [113] developed photo-activated nanoliposomes that, after a light-initiated and rapid release of antitumor drug doxorubicin, imparted cytotoxicity and reversal of drug resistance. All these recent publications indicate that the specific targeting of anthracyclines to tumor tissues could be a winning strategy to overcome drug toxicity and improve efficacy.

## 6. Interactions of Anthracyclines with the Immune System

Historically, the idea of combining immunotherapy and chemotherapy was proposed soon after daunomycin discovery, as parent drugs daunomycin and adriamycin (Figure 1) were observed to synergize with the immune viral response in a mice model of Moloney virus-induced tumors [114,115]. However, these lines of investigations were abandoned during the next decades as anthracycline-containing chemotherapies were considered to kill proliferating tumor cells, via apoptosis or necrosis, in a manner independent from the immune system of patients. However, this common thought has changed in recent years as, for instance, murine tumors are much more sensitive to anthracyclines when they grow in syngenic immunocompetent than immunodeficient animals [116].

Several studies have established that anthracyclines can have cytostatic effects and can lead cells not only to apoptosis, but also to cell senescence or other types of death programs, including immunogenic death [117,118]. Anthracycline-induced (and more generally, DNA damage-induced) senescence is not due to telomere shortening and cannot be rescued by telomerase expression, which are instead molecular characteristics of normal cell proliferation limits. Terminal proliferation and senescence were shown to be induced by low-cytotoxic doses of doxorubicin in a number of cancer cell lines [117]. The terminal arrest was dependent on p53 and p16^INK4a^ tumor suppressor genes and occurred within a few cell cycles from doxorubicin treatment. Interestingly the authors also described the occurrence of micronuclei formation promoted by the drug, which was associated with mitotic death [117]. Even if senescent cells cannot proliferate, they are still metabolically active, and can therefore interact with other normal or cancer cells in patients. How this can affect the anticancer activity of anthracyclines needs to be better understood.

The mechanistic ways a cancer cell can die upon treatment with chemotherapeutics constitute an active research area. Cell death programs appear to be diverse and therefore the definition of the nature of cell death under any condition has become complex. Currently, a consensus has emerged recommending that a cell death should be defined by distinct molecular, biochemical, metabolic, and morphological hallmarks [119,120,121]. Interestingly, a new cell death modality has been recognized: the immunogenic cell death (ICD) that is characterized by dead-cell antigens released by dying cells and able to elicit specific immune responses against cancer cells [117,118]. This pathway utilizes signals emanating from dying cancer cells to inhibit cancer growth and was first defined in a context of anti-cancer chemotherapy using anthracyclines [116]. A comparison of doxorubicin and mitomycin C (a doxorubicin-unrelated agent) showed that doxorubicin-induced dying cells were immunogenic in a variety of cancer cell types, whereas dying cells due to mitomycin C were not. Caspase activation was also a determinant of immune response as inhibition of caspase fully abolished the immunogenicity of doxorubicin-induced dead cells. However, as caspase was also activated in mitomycin C treated cells, it was proposed that specific factors were present only in doxorubicin-treated cells, which however remained to be established [116]. Further analyses have shown that specific aspects of immunogenic cell death are the early exposure of reticular chaperone calreticulin on the dying cell surface, the late release of the nuclear non-histone protein HMGB1 (high mobility group B1), and the secretion of ATP extracellularly. These factors are recognized by specific receptors of dendritic cells (DCs) which then activate an immune response against cancer cells [118,122]. Interestingly, autophagy is required for optimal ATP release but dispensable for the emission of other immunogenic signals [123]. This has a consequence on drug activity as autophagy is dispensable for mitoxantrone-induced cell death—mitoxantrone shares the antraquinone planar ring moiety with anthracyclines—but required for its immunogenicity. The findings indeed showed that autophagy can contribute to the drug therapeutic effects by provoking an anticancer immune response [123].

However, the effects of anthracyclines, and other chemotherapeutics, on cancer cells can even be more complex as drugs can also enhance the expression of specific interferon genes directly in treated cancer cells that then stimulate the innate immunity of the host. In the later 1990s, doxorubicin and other DNA damage agents were shown to activate IRFs (interferon regulatory factors) in human cancer HeLa cells [124,125]. IRFs have protective functions in cell defense mechanisms against environmental stresses, including viruses and bacteria. In particular, doxorubicin activates IRF1 gene expression while increasing the phosphorylated form of IRF3, suggesting that IRF1 and IRF3 may work in a protective pathway constituted by an immediate IRF3-dependent response and a later IRF1-dependent phase. IRF1 can modulate specific target genes, thereby inducing cell cycle arrest, while IRF3 is phosphorylated during viral infections and translocates in the nuclear compartment to activate the transcription of target genes [124,125]. More recent papers have shown that IRF3 is involved in the STING pathway that activates the transcription of type I interferons upon the occurrence of cytoplasmatic nuclei acids [126]. The IRF protective pathways lead to transcriptional activation of genes with antiproliferative, apoptotic, antiviral, and immuno-stimulatory activities. Therefore, anthracycline-induced IFN responses and immune system stimulation may contribute to the antitumor activity of doxorubicin [127,128].

Interestingly, it has become clear that several molecular features of immune response to viral infections are similar to successful anticancer chemotherapies based on anthracyclines. Such ‘viral mimicry’ has been even suggested to be a hallmark of clinically-effective chemotherapy [129]. Anthracyclines can activate the expression of type I interferons by an autocrine and paracrine circuitry, which depends on TLR3 (Toll-like receptor 3) and TLR4 signaling and release of RNA during cell death. A retrospective analyses of cohorts of patients with breast cancers showed that high expression of type I Interferon gene signature predicts the response to anthracycline-based chemotherapy in subsets of patients [129]. These findings may open up individualized therapy based on immunological characteristics of patient tumor and the targeted delivery of type I interferon in patients with low interferon expression.

More recently, from a screening of 2080 bioactive compounds, five agents, including four anthracyclines, were shown to induce the IFN response in an ATM kinase-dependent manner upon Ebola virus infection of human cells. The compounds activated the STING pathway of IFN induction resulting in the inhibition of virus replication [130]. Interestingly, viral proteins, which can effectively inhibit host immune-response genes, could not prevent IFN response by doxorubicin. Overall, the report suggests that doxorubicin-mediated IFN activation may be due to two distinct, ATM- or STING-dependent pathways. Another recent paper on hepatitis B virus (HBV) showed that daunorubicin can trigger innate immune response in a cGAS-dependent manner preventing HBV production in human hepatocytes [131]. Therefore, overall these results support the fact that anthracyclines can activate the STING-cGAS pathway activating type I interferons in cancer cells.

## 7. Conclusions

Sixty years since the discovery of daunorubicin [1], this class of antitumor antibiotics is still an essential component of effective chemotherapeutic regimen of solid and hematopoietic cancers. While the tremendous efforts of the past decades aimed at the discovery of more effective analogs have substantially failed, major advances in cancer genetics can now provide precise genetic information for selecting patients that will gain the most from a treatment with anthracyclines. At the same time, the more recent developments of very effective immunotherapies of solid tumors open up the possibility of improved combinations of immunotherapy and chemotherapy. The appreciation of the contribution of the immunological system in the anticancer activity of anthracyclines has revealed unexpected immunological effects induced by these drugs. Interestingly, the effects are not specific for anthracyclines, as they have been reported to occur also for other, but not all, anticancer agents such as cisplatin derivatives and ionizing radiation. A common feature of these agents is the ability to damage the DNA, however the mechanistic link between DNA damage and the immune response is not yet clear. In particular, the molecular mechanisms connecting the anthracycline targets (Top2α and Top2β), in the nucleus/mitochondria, to the STING-cGAS pathway, in the cytoplasm, remain to be fully established. We expect that the complete definition of structural aspects and molecular pathways of the interaction of anthracycline with the immune system may open up new opportunities of clinical utility of these Top2 poisons.
